# Rebuttal to: ‘Gender affirming healthcare’ is not what the family physician needs to know

**DOI:** 10.4102/safp.v67i1.6185

**Published:** 2025-09-05

**Authors:** Elma de Vries, Madeleine Muller, Anastacia Tomson, Chris McLachlan

**Affiliations:** 1 School of Medicine, Faculty of Health Sciences, Nelson Mandela University, Gqeberha, South Africa; 2 Department of Family Medicine and Rural Health, Faculty of Health Sciences, Walter Sisulu University, East London, South Africa; 3 Department of Family Medicine, Cecilia Makiwane Hospital, East London, South Africa; 4 My Family GP, Cape Town, South Africa; 5 Department of Psychology, Faculty of Social Sciences, University of South Africa, Pretoria, South Africa

As authors of a peer-reviewed article published in this journal,^1^ we would like to thank the editors for the opportunity to respond to the letter by Giddy et al.,^2^ which criticised the premise of gender-affirming healthcare (GAHC) as outlined by the Southern African HIV Clinicians Society (SAHCS) Guideline.^3^ While we agree with the importance of an evidence-based approach, several claims in the letter require clarification and rebuttal.

## On the title and ethos of family medicine

The view expressed in the letter is fundamentally at odds with both the principles and practice of family medicine. The national position article on the contribution of family physicians (FPs) to district health services^4^ affirms that FPs ‘are committed to the biopsychosocial approach and person-centred care’. It highlights their responsibility to respond to changing health needs, improve access to care, and implement innovation. Our article^1^ was aligned with this ethos and sought to support FPs in providing affirming, evidence-based care to transgender and gender diverse (TGD) patients.

McWhinney,^5^ widely regarded as a foundational thinker in family medicine, stated that ‘the family physician is available for any health problem’, and that it is the patient who defines the problem.^5^ If a patient presents with gender dysphoria and seeks support, this is unquestionably within the scope of family medicine.

While McWhinney^5^ wrote from a Canadian perspective, Ras^6^ writes about an African re-imagination of the doctor–patient relationship from an *Ubuntu* perspective. He describes this re-imagination where patients are empowered, engaged agents, who are ‘affirmed in their personhood and patient-hood by a close attention to their suffering’. Furthermore, a 2024 article^7^ on sexual health in primary care in this journal underlined the duty of health professionals to ensure that every patient is affirmed, respected, and not judged, including TGD individuals. It concluded that primary care practitioners play a crucial role in addressing patients’ sexual health concerns.^7^

To suggest, as the title of the letter does, that FPs need not be equipped to care for TGD patients, undermines both the ethos of family medicine and the ethical obligations of healthcare practitioners. The Health Professions Council of South Africa’s ethical guidelines^8^ explicitly affirm the rights of patients to self-determination and to make informed choices based on their beliefs and values.

The letter^2^ argues that GAHC should not happen at any level of care, ‘let alone in the primary health care setting’. We disagree. Although not all components of GAHC would be for primary care, the SAHCS guideline^3^ states that hormone therapy can be provided at primary care for adults, with referral to an endocrinologist for complex cases. Our article^1^ stated that its focus will be ‘only on informed consent in the adult patient for hormone therapy’. Gender-affirming healthcare for youth requires a multidisciplinary team that includes a mental health practitioner.^3^

## Terminology and scientific accuracy

The letter^2^ challenges the terminology used in our article, yet employs terminology such as ‘gender distress’ and ‘transgender ideation’ that are not used in peer-reviewed publications on GAHC and appear to question the validity of transgender identities. Such language pathologises gender diversity and contributes to stigma.

Referring to intersex in quotation marks is also deeply problematic and invalidating. The term intersex is an established scientific descriptor of variations in sex characteristics, including chromosomes, hormones, and reproductive anatomy.^9^ The lived experiences of intersex people should not be dismissed.^10^ Many intersex people have experienced medical trauma through violation of bodily autonomy, and as health professionals, we are called upon to educate ourselves on the needs of intersex people.^10^

We are also concerned by the use of sensationalist and inaccurate language such as ‘breast and penis amputations’ to describe gender-affirming surgical interventions. These procedures are accurately described in medical literature as masculinising chest surgery^11^ or vaginoplasty^12^ and should be referred to with appropriate clinical terminology. Research into the use of stigmatising language in scientific literature has led to recommendations that authors and publishers have a responsibility to promote the use of non-stigmatising language in HIV and other health conditions.^13^

## On referencing and evidence

The letter refers to and quotes from the Cass review.^14^ This review has been criticised by several authors for methodological aspects,^15,16,17,18^ with more recent guidelines making recommendations based on the best available evidence.^15,19^

The assertion that a systematic review by Baker et al.^20^ ‘showed no evidence for efficacy and safety’ is misleading. The authors, in fact, found that gender-affirming hormone therapy ‘was associated with increased quality of life, decreased depression, and decreased anxiety’.^20^

The letter further claims that an independent review^21^ concluded the SAHCS Gender-Affirming Healthcare Guideline ‘should not be followed’. This is inaccurate. The cited review^21^ applied the AGREE II tool to assess 23 guidelines globally and did not single out any for dismissal. Rather, it found that none scored above 70% across all six appraisal domains, which reflects a call for stronger guideline development across the field, not a discrediting of any particular one.

## Factual inaccuracies in the letter

Several statements in the letter are factually incorrect:

- The claim that GAHC includes ‘puberty suppression in prepubertal children’ is false. Guidelines^3,22^ clearly state that no medical interventions are provided to prepubertal children. Puberty suppression can be considered by a multidisciplinary team once Tanner Stage 2 of puberty has been reached.^3,22^
- The assertion that GAHC creates a ‘lifetime dependency on the health system in people with previously healthy bodies’ ignores the psychosocial burden of untreated gender incongruence and disregards mental health outcomes. This reductionist view undermines the biopsychosocial model that FPs are committed to.^4^
- The statement that the National Department of Health has no policies on GAHC is incorrect. The *National Strategic Plan for HIV, TB and STIs 2023–2028*^23^ mentions transgender 28 times and explicitly calls for the ‘inclusion of gender affirmation package of services at all levels of care’. In 2019, the South African National Essential Medicines List Committee approved gender-affirming hormones for tertiary level of care.^24^
- The assertion that GAHC places young people ‘at risk of serious harm’ is unsubstantiated and not supported by the evidence. On the contrary, withholding care is associated with increased risk of mental health problems, including suicidality.^25^ The systematic review by Baker et al.^20^ referenced in the letter reported that ‘no studies showed that hormone therapy harms mental health or quality of life among transgender people’.

We agree that clinicians need to follow standard and accepted medical and psychological approaches and contend that GAHC is accepted practice that is evidence-based.^26^ Recent French^19^ and German-Austrian-Swiss^15^ guidelines provide clear guidance on the ethics and approach to GAHC.

## Conclusion

In accordance with the values and responsibilities of family medicine, we assert that it is necessary to equip FPs with the knowledge and skills to provide GAHC. This is essential to the realisation of equitable, person-centred, and dignified healthcare for all.
